# COVID-19 Vasculitis and vasculopathy-Distinct immunopathology emerging from the close juxtaposition of Type II Pneumocytes and Pulmonary Endothelial Cells

**DOI:** 10.1007/s00281-022-00928-6

**Published:** 2022-04-12

**Authors:** Sami Giryes, Nicola Luigi Bragazzi, Charles Bridgewood, Gabriele De Marco, Dennis McGonagle

**Affiliations:** 1 Leeds Institute of Rheumatic and Musculoskeletal Medicine (LIRMM), University of Leeds, Leeds, UK; 2 National Institute for Health Research (NIHR) Leeds Biomedical Research Centre (BRC), Leeds Teaching Hospitals, Leeds, UK

**Keywords:** SARS-CoV-2, COVID-19 Vaccine, Vasculitis, Vasculopathy, Endotheliitis, Immunothrombosis

## Abstract

The SARS-CoV-2 virus ACE-2 receptor utilization for cellular entry and the defined ACE-2 receptor role in cardiovascular medicine hinted at dysregulated endothelial function or even direct viral endotheliitis as the key driver of severe COVID-19 vascular immunopathology including reports of vasculitis. In this article, we critically review COVID-19 immunopathology from the vasculitis perspective and highlight the non-infectious nature of vascular endothelial involvement in severe COVID-19. Whilst COVID-19 lung disease pathological changes included juxta-capillary and vascular macrophage and lymphocytic infiltration typical of vasculitis, we review the evidence reflecting that such “vasculitis” reflects an extension of pneumonic inflammatory pathology to encompass these thin-walled vessels. Definitive, extrapulmonary clinically discernible vasculitis including cutaneous and cardiac vasculitis also emerged- namely a dysregulated interferon expression or “COVID toes” and an ill-defined systemic Kawasaki-like disease. These two latter genuine vasculitis pathologies were not associated with severe COVID-19 pneumonia. This was distinct from cutaneous vasculitis in severe COVID-19 that demonstrated pauci-immune infiltrates and prominent immunothrombosis that appears to represent a novel immunothrombotic vasculitis mimic contributed to by RNAaemia or potentially diffuse pulmonary venous tree thrombosis with systemic embolization with small arteriolar territory occlusion, although the latter remains unproven. Herein, we also performed a systematic literature review of COVID-19 vasculitis and reports of post-SARS-CoV-2 vaccination related vasculitis with respect to the commonly classified pre-COVID vasculitis groupings. Across the vasculitis spectrum, we noted that Goodpasture’s syndrome was rarely linked to natural SARS-CoV-2 infection but not vaccines. Both the genuine vasculitis in the COVID-19 era and the proposed vasculitis mimic should advance the understanding of both pulmonary and systemic vascular immunopathology.

## Introduction- The Scope of SARS-CoV-2 Related Vascular Pathology

The novel highly transmissible SARS-CoV-2 virus resulted in fatal pneumonia in a subset of patients and quickly garnered great interest in the cardiovascular and rheumatology arenas, because of the prominent vascular immunopathology. The most striking pathological feature was extensive viral alveolitis but also vascular thrombosis and reports of vascular wall inflammation [1, 2]. Compelling evidence for extrapulmonary vasculitis and vasculitis mimics also emerged during the COVID-19 pandemic which are described in in this article. We also review the extant literature by performing a systematic literature review up until September 2021 that has reported genuine vasculitis in SARS-CoV-2 infection, and we also review post-COVID-19 vaccinations for an emergent vasculitis signal given that most available SARS-CoV-2 vaccines are directed to the spike protein which engages the ACE2 receptor that is known to be expressed on endothelial cells[3]. We also cover the totality of systemic vascular complications—whether vasculopathy, vasculitis or vasculitis mimics and describe how these appear to be independent of productive infection of vascular endothelial cells. We will also focus on extrapulmonary vascular pathology including genuine autoimmune vasculitis (referred also as true, bespoke, or bona fide vasculitis in this text) and a novel potential vasculitis mimic related to diffuse immunothrombosis outside the pulmonary territory that has been reported in SARS-CoV-2.

## ACE2 Centric Vasculopathy model including vascular infection

The SARS-CoV-2 virus employs the ACE2 receptor for cellular entry with this receptor having a widespread upper and lower respiratory tract distribution from nasal epithelium to the alveoli, most notably alveolar type 2 pneumocytes[4, 5] but the ACE2 receptor also has a well-established role in the cardiovascular system[6, 7]. Also, the comparatively minuscule SARS epidemic at the turn of the millennium had already provided substantial information about this related structurally close beta-coronavirus that also used the ACE2 receptor [4]. Beyond the lower respiratory tract symptoms of COVID-19 pneumonia explained by ACE2 receptor expression, upper respiratory tract symptoms of anosmia and pharyngitis could also be linked to high local ACE2 receptor expression levels[8]. Prior data also showed ACE-2 receptor expression on other cell types including endothelial cells and cardiomyocytes[4, 9] and provided potential pointers to the novel emergent cardiovascular pathology in the initial COVID-19 wave. Independently, of these observations in SARS, the role of ACE2 receptor as a regulator of cardiovascular system including hypertension, myocardial injury, obesity and diabetes was reported in several models systems, but to variable extents[10, 11]. Cardiac enzyme elevations and lung and systemic vasculopathy quickly cemented the notion that ACE-2 dysfunction in the cardiovascular system was a key mortality driver in emergent COVID-19 pneumonia and associated vascular pathology [12, 13]. Accordingly, early in the pandemic, there was great interest in the influence of cardiovascular drugs that modulated ACE2 expression including Renin–Angiotensin inhibitors, nonsteroidal anti-inflammatory, and many others [3, 14–16].

The prior SARS epidemic was associated with both pulmonary capillary and larger vessel thrombosis and also reports of viral myocarditis and in common with SARS-CoV-2, the SARS virus also utilized the ACE2 receptor [17–19]. Given that recombinant ACE-2 mitigates against experimental pneumocyte injury, and since SARS-CoV-2 spike protein can downregulate ACE-2[20], it has been considered that SARS-CoV-2 derived spike protein without actual infection might trigger endothelial cell dysregulation and immune activation[21]. A third beta coronavirus termed Middle East respiratory syndrome coronavirus (MERS-CoV) has been another twenty-first century emergent coronavirus and limited pathological literature supports the idea of an identical immunopathology with pneumonia and reported immunothrombosis and an increased mortality in subjects with cardiovascular risk factors[22–24]. As this latter virus shows an identical immunopathology but does not target the ACE2 receptor [23, 25] and recent studies showing low or even absent endothelial cells ACE2 expression [26–28], then it is likely that novel beta coronaviruses are capable of mediating immunopathology, including vasculitis, independently of the ACE-2 receptor and that factors extrinsic to ACE-2 appear to be critical to COVID-19 related vasculopathy, which is the focus of this paper.

## Histological Reports of Pulmonary Vasculitis in COVID-19 and what it means

**Fig. 1:**
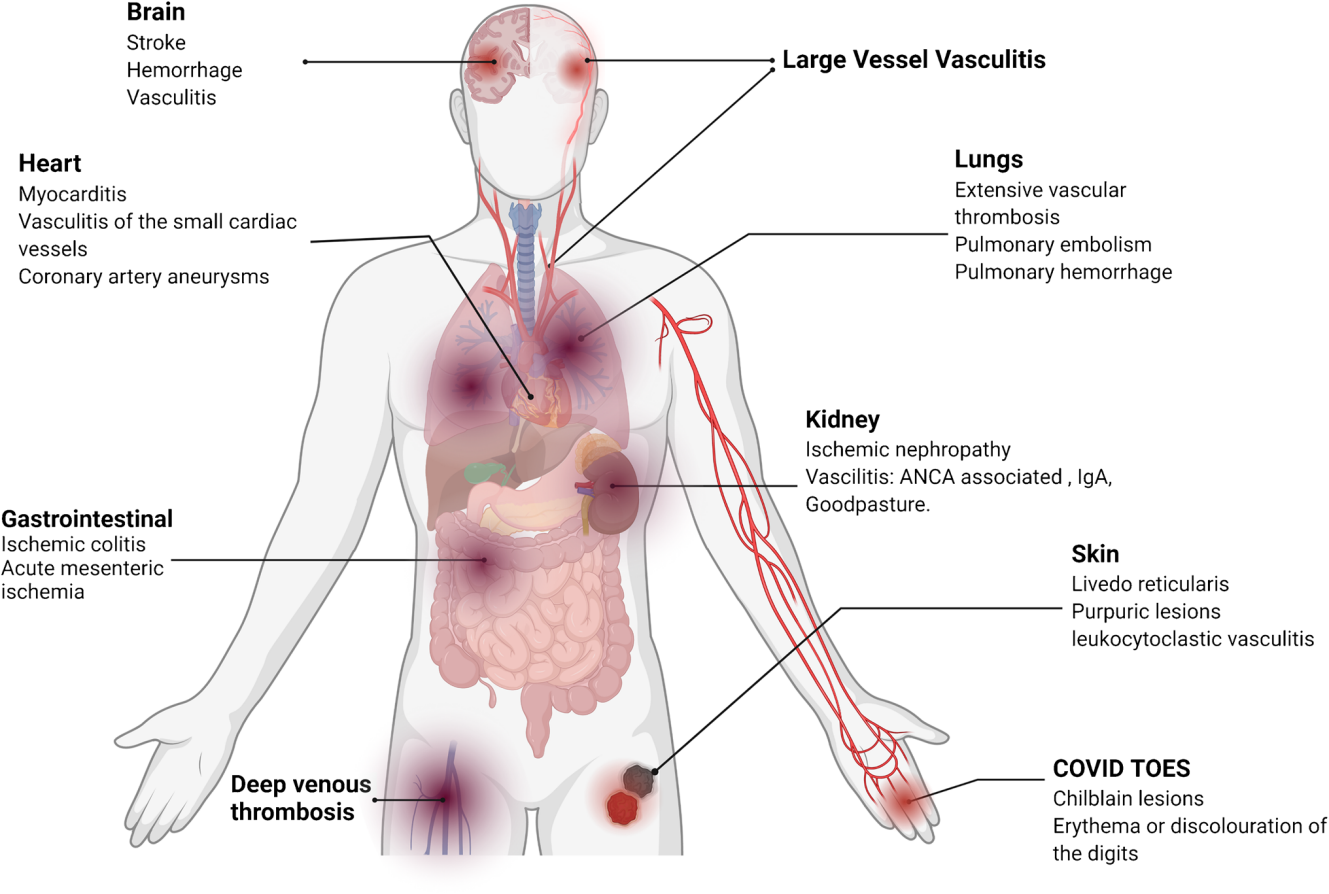
The many faces of COVID-19 vasculopathy and vasculitis

Pathology from the primary lung target organ in COVID-19 have consistently shown that viral alveolitis is accompanied by peri-capillary myeloid and lymphoid cell infiltration [29, 30]. Pulmonary capillary, pulmonary arteriolar and pulmonary venular vessel wall inflammation has been associated with extensive and pervasive vascular luminal thrombosis[2, 31]. Initially, it was considered that endothelial inflammation due to direct viral infection or “viral endotheliitis” accounted for the immunothrombosis [29, 32]. However, it has since emerged that endothelial cells have comparatively low ACE2 expression and are also fairly resistant to productive viral infection supporting the concept that a “genuine vasculitis” rather than active infection, may thus account for the SARS-CoV-2 vascular pathology (Fig. 1) [26–28]**.** Indeed, it has likewise emerged that direct endothelial infection with productive SARS-CoV-2 viral replication does not occur in humans [27, 33, 34]. Also, in the prior SARS outbreak, reports of direct endothelial infection were never conclusively shown [35, 36].

**Fig. 2:**
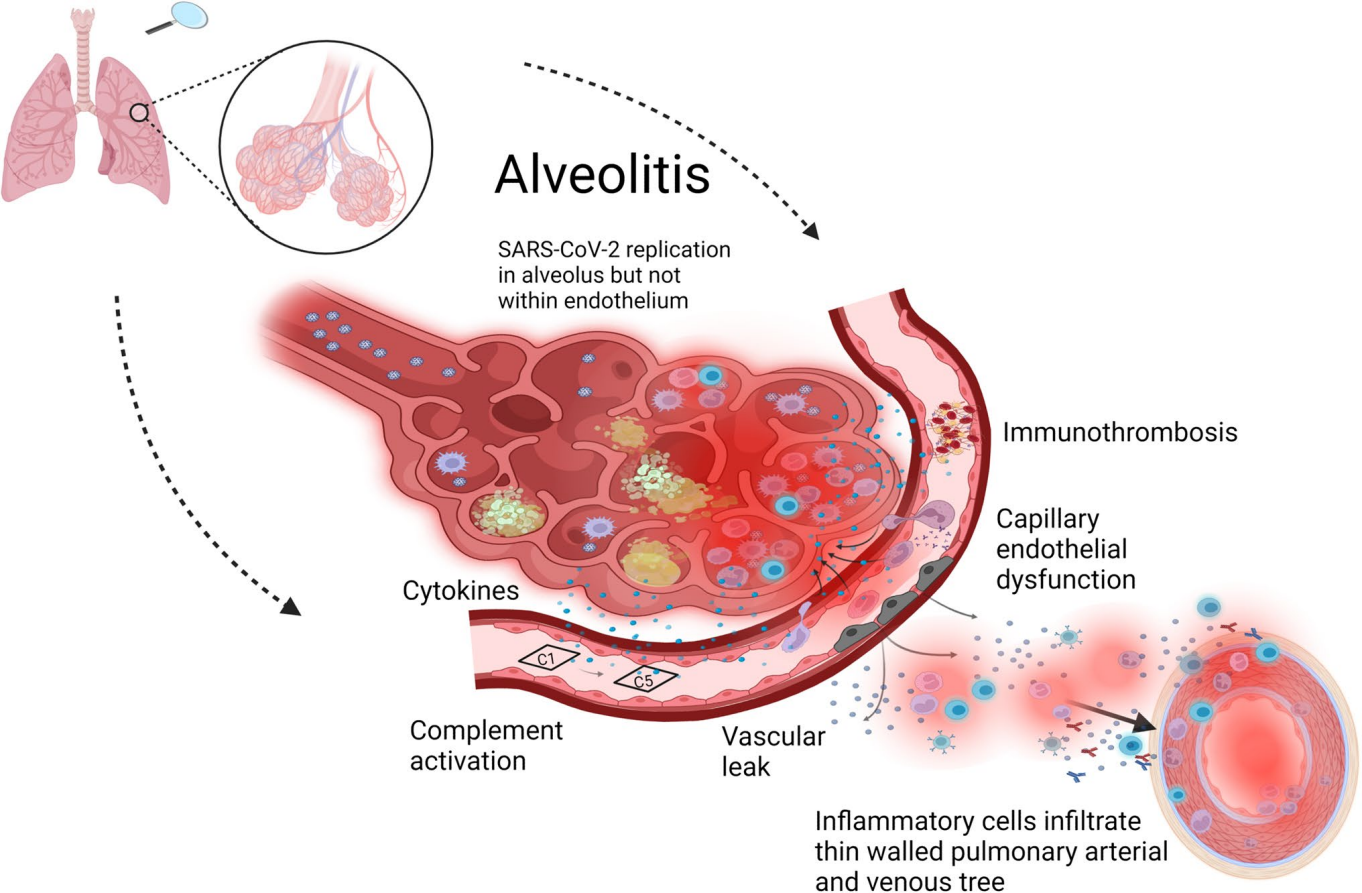
Pulmonary vascular changes reminiscent of vasculitis in severe COVID-19. Why does COVID-19 lung disease exhibits endothelitiis and vascular inflammation but is not a genuine vasculitis? Primary alveolitis with an influx of neutrophils, macrophages and T cells in severe COVID-19 triggers activation of the immediately adjacent endothelium of the capillaries. Inflammation triggered endothelial damage is associated with immunothrombosis via tissue factor and other mechanisms. Tissue destruction, hypoxia and viral PAMPS also activate complement in the capillary environment. The pulmonary venular and arteriolar vessels are thin-walled and closely juxtaposed to the inflamed alveolar network, which results in immune cell infiltration of the vascular wall contributing to vasculitis like histology. However, there is no compelling evidence for direct endothelial infection

Pathological pulmonary changes described as vasculitis have been reported in COVID-19 pneumonia. In one study, around a quarter of subjects had perivascular lymphocyte cuffing or capping, that was described as compatible with vasculitis[31]. In another postmortem study, 4 of 11 cases had predominant macrophage infiltration into the pulmonary arterial wall, and also CD4, CD8 T-cells and B cells were reported, with these histological features being designated as arteritis[37]. One more pathological report described an endarteritis obliterans in conjunction with C5-9 complement pathway activation at the site of vasculitic change[38]. However, the pervasive impact of severe alveolitis with associated inflammation in the juxta-capillary and thin-walled pulmonary vascular system may account for the extensive lung vascular pathology (Fig. 2). We suspect that studies demonstrating juxta-capillary lymphocytic infiltration that have been attributed to a “viral endotheliitis” may reflect lymphocyte migration or infiltration either to or from the closely juxtaposed pneumonic alveolar territory (Fig. 2) [1, 29, 39]. Rather than representing a primary pulmonary vasculitis in the later phases where the SARS-CoV-2 virus is cleared, such microscopic “vasculitis” may be part of the so-called Virchow's triad where a severe extra-vascular alveolitis leading to vascular wall inflammation is linked to vessel wall immune cell infiltration and luminal thrombosis (Fig. 2).

Severe alveolar centric inflammation without actual endothelial infection likely leads to extensive immunothrombosis by a myriad of mechanisms including pathogen-associated molecular patterns (PAMPs) and damage-associated molecular patterns (DAMPs) related endothelial activation[40, 41]. Factors including tissue factor released by activated immune cells in the lungs or systemically also likely trigger coagulation[40]. Viral RNA access to the capillary lumen consequent to damage of the sub-micrometer alveolar–capillary barrier may also contribute to Factor XII and X activation with local coagulation cascade activation [42–44]. Presently, the understanding of the COVID-19 lung vascular immunopathology is conceptualized in terms of severe immunothrombosis that constrains SARS-CoV-2 to the alveolar territory[45, 46]. The severity and magnitude of the alveolar centric inflammation leads to profound endothelial cell damage and even endothelial cell death captured under the umbrella term of endothelialopathy[47, 48].

## What Did We Know Before The COVID-19 Era About Infection and Vasculitis?

The question of vasculitis as a manifestation of infection disease is not a unique to COVID-19 and was well described phenomenon with the oldest pathogens know for humankind such as syphilis and tuberculosis. In most of the cases, this infectious vasculitis is a consequence of direct invasion, extension of localized focus or septic embolization of bacterial, viral, fungal, or parasitic infections to the endothelial cells and vascular wall which is usually accompanied by intense inflammatory response to the vessel wall and other symptoms related to the main infection and rarely confused with bona fide vasculitis [49, 50]. For example, in syphilis, treponema pallidum invasion of endothelial cells and endothelial barriers such retina, placenta, and blood–brain barrier is believed to be one of the main bacteria virulence factors; hence direct syphilis infection may cause different vasculitic syndromes from central nervous system vasculitis, retinal vasculitis and aortitis [51–53]. Other pathogens have been studied as a possible mechanistic triggers in the development of bona fide vasculitis, such as staphylococcus aureus in ANCA associated vasculitis and streptococcal infection in Henoch–Schonlein purpura (HSP), but the causality in these cases is still controversial[54, 55]. Only for a few pathogens has causality been established, such as Hepatitis C Virus (HCV) related cryoglobulinemic vasculitis, which is mediated mainly by the binding of HCV viral particles to IgM with rheumatoid factor activity resulting in the production of cold-precipitable immune complexes, which binds to endothelial cells activating the complement system and inflammatory response [56–58].

Vaccinations including influenza vaccine, hepatitis B, Bacille Calmette-Guerin (BCG), and human papillomavirus vaccines, all of which lack live microbes with the exception of BCG, have been associated with occasional vasculitis development [59]. The commonest patterns of vasculitis from the same study were HSP and Kawasaki disease. As most of these vaccines do not contain viable replicating pathogens, these findings suggest a non-specific activation of immunity in susceptible individuals that results in vasculitis.

## COVID-19 Pulmonary Pathology versus Behcet's Pulmonary Vasculitis

It is useful to compare the COVID-19 pulmonary vasculopathy with the genuine pulmonary vasculitis in Behcet's Disease (BD) that is usually characterized by an absence of interstitial pathology but pulmonary vascular wall inflammation with neutrophilic inflammation and aneurysmal dilatation[60]. The BD vascular centric inflammation is associated with both superficial and deep immunothrombosis and neutrophilic inflammation[60, 61]. Likewise, neutrophils play a key role in the immunothrombosis associated with COVID-19 with netotic material, platelets and other immune cells forming a key part of the clot in COVID-19 disease [62, 63]. Moreover, in critical COVID-19 patients there is evidence of extensive immune cell activation in the peripheral blood that also includes neutrophils[64].

An interesting facet pointing to shared features between BD and COVID-19 pulmonary vascular involvement is anti-coagulation inefficacy in BD immunothrombosis and likewise in critical COVID-19 pneumonia where full dose anti-coagulation has no benefit and may be potentially detrimental[65]. Therapeutically, corticosteroids may be beneficial in deep venous thrombosis (DVT) and other severe manifestations of BD as in severe COVID-19 patients [66]. Although an element of pulmonary haemorrhage is a histological feature of severe COVID-19, this may be dysregulation perfusion rather than the aneurysmal rupture as seen in BD. In keeping with the fact that the genuine vasculitis associated with BD is completely distinct from the COVID-19 immunopathology is the limited data suggesting exacerbation of BD related vascular pathology being linked to COVID-19 infection or vasculitis. In one report, of ten patients with BD during COVID-19 infection, one patient with long-standing BD and central nervous system involvement reported having a DVT, which was unusual to his BD course and responded to steroids without anticoagulation [67]. We have noted occasional BD flares following SARS-CoV-2 vaccination, but these were restricted to mucocutaneous disease and arthritis[68].

## Bona Fide Vasculitis in COVID-19

We performed two separate systemic literature reviews of COVID-19 related vasculitis using the following search engines; PubMed/MEDLINE, Scopus, Google Scholar and Embase. The searches were conducted to include articles until September 2021. In the first search, we looked for reported cases of vasculitis after SARS-CoV-2 infection and the second after COVID-19 vaccination. We used this strategy as we reasoned that systemic infection with severe lung damage may predispose to vasculitis compared to the controlled inoculum strategies of vaccination, where the lung tissue is spared severe damage.

For this purpose, we used the following search string in the first search: (SARS-CoV-2 OR COVID-19 OR "novel Coronavirus" OR "emerging Coronavirus") AND (vasculitis OR "Giant cell arteritis" OR Takayasu OR “large-vessel vasculitis” OR Cogan OR "Polyarteritis nodosa" OR Buerger OR "ANCA-associated vasculitis" OR (“antineutrophil cytoplasmic antibodies” AND vasculitis) OR Wegener OR polyangiitis OR Churg-Strauss OR "Urticarial vasculitis" OR Goodpasture OR “anti-glomerular basement membrane disease” OR “cutaneous small-vessel vasculitis” OR “cutaneous vasculitis” OR “IgA vasculitis” OR “leukocytoclastic vasculitis” OR “Henoch-Schönlein Purpura”).

For the second search strategy in SARS-CoV-2 vaccinated subjects, we used the same search string, but we added the vaccine-related component: (vaccine OR vaccination OR immunisation OR immunization). Medical subheadings (MeSH) terms and wild card truncated words option were used when necessary.

Clinical case reports and case series providing sufficient details were included (for instance, case reports/case series reporting only clinical illustrations/image were excluded). Reports of vasculitis like features including thrombosis and associated complement activation within vessels were not defined as vasculitis but considered as immunothrombotic vasculitis mimics and were not included in the vasculitis groups. Articles were excluded if the vasculitis could be linked to a drug, rather than directly to the SARS-CoV-2 infection or vaccination or if the vasculitis relapsed and not newly diagnosed. Epidemiological surveys computing incidence/prevalence rates but not providing sufficient clinical details were not retained in the analysis. Extensive cross-referencing was applied, to ensure the maximum coverage and to increase the chance of including all relevant articles. Already existing reviews (in particular, systematic reviews) were scanned to include already abstracted data and eventually update them. We did not search for “COVID toes” or Kawasaki Disease (KD) that is linked to Multisystem Inflammatory Syndrome (MIS). Our focus was on previously known systemic vasculitides rather than the two recently defined vasculitis entities.

## Vasculitis in SARS-CoV-2 Infection

Excluding Kawasaki disease like vasculitis associated with the MIS spectrum and “COVID toes”, only forty-eight vasculitis cases were described (Table 1) [69–94]. These are described starting with large vessel vasculitis.

**Table 1:** Reported cases of genuine vasculitis in SARS-CoV-2 infection

	N.of cases	Gender	Age	Underlying conditions	Symptoms	Workup	Timing	Treatment	Outcome
Cutaneous: CSVV /LCKV[69–75] / UV[76–82]	**CSVV/LCKV**:6 **UV**: 9	7 X M 8 X F	1.5–83	Only in three patients: 1. HTN, TIA, AF, CKD 2. HTN; DM 3. HTN, MI, HF, COPD One pregnant (a case of UV)	**CSVV/LCKV**: chilblain-like, ulcerative lesions; arthralgia; constitutional symptoms **UV**: Urticarial rash; pruritic rash; constitutional symptoms; arthralgia; abdominal tenderness; conjunctival erythema with periorbital edema; acral nonpitting edema; diarrhea; hypotension	Clinical; Neutrophilia; Lymphopenia; anemia hypoalbuminemia with albuminuria; C3-; LDH + CRP + ESR + ; Cr + ; auto immune serology + ; SARS-Cov-2 serology; skin biopsy; immunolabelled SARS-CoV-2 antigens in skin biopsies;chest CT scans/ HRCT	5 days- 3 weeks after COVID-19 symptoms onset; sometimes, COVID-19 diagnosed after vasculitis onset	**CSVV:** Paracetamol; steroids **UV:** Antihistamines, steroids, Colchicine, HCQ, heparin if needed	Two deaths (a case of UV and a case of CSVV/LCKV)
ANCA-associated vasculitis[83–87]	6	2XF 4XM	25–64	Only in Two patients: 1.DM 2.DM and scleroderma	Fever, respiratory symptoms; GI symptoms	Clinical; ANCA + (MPO in 3 cases, PR3 in 3 cases); Cr + ; Proteinuria; Haematuria; Skin Biopsy; Renal biopsy; Chest CT scan	Simultaneously or shortly after COVID-19 diagnosis	Glucocorticoids; CYC; PEX; HCQ; rituximab, if needed	Improvement / Resolution Some with end organ damage
IgAV/HSP[88–90]	16	13XM 3XF	~ 1–78	HTN; Alcohol consumption; HyperL; aortic Stenosis; bladder cancer; Hirschprung disease; Crohn disease; none or not reported in 10 cases	Fever; rhinorrhoea, cough; chills; dyspnea; myalgia, fatigue; headache; pruritic rash; maculopapular rash, arthralgia or arthritis; GI symptoms (nonbloody diarrhea, hematochezia, vomiting); lower limbs pitting edema; HTN	Clinical; CRP + ; ESR + ; C3-; leucocytosis; Anaemia; thrombocytosis or thrombocytopenia; albumin-; proteinuria; haematuria; Cr + ; hyaline casts; renal biopsy and electron microscopy; skin biopsy; IF	Simultaneously-37 days after COVID-19	Steroids; ABX; anticoagulation; antivirals; NSAIDS; statins; Rituximab, anti-helminthics	One patient died
LVV/GCA[91, 92]	2	2XM	47–50	None	Headache; temporal thickening; paracentral acute middle maculopathy in one case	Clinical; Doppler ultrasound of the right temporal artery; FDG PET-CT scan	Simultaneously (one case); 2 months after COVID-19	N/A in one case; Steroids in the second case	Full resolution
Goodpasture[90, 93, 94]	9	2XM 7xF	27–73	HTN; rheumatic HD; COPD; SLE; asthma; bronchiectasis; None in two cases	Fever; fatigue; myalgia; GI symptoms; haemoptysis; epistaxis; petechial rash; dyspnoea; ARDS	Clinical; WBC + , lymphocytes-, Cr + and BUN + ; CRP + , ESR + ; proteinuria; chest CT scan	Simultaneously	ABX; steroids; CYC; Rituximab; PEX; Haemodialysis	One patient died

Abbreviation: CSVV- Cutaneous small-vessel vasculitis, LKCV- Leukocytoclastic vasculitis, UV- Urticarial vasculitis ,IgAV- IgA Vasculitis, HSP- Henoch-Schönlein Purpura, ANCA -Antineutrophil cytoplasmic antibodies, LVV – Large vessel vasculitis , (+) - positive/elevated, (-)- Decrease, F- Female, M- Male, HTN- Hypertension ,HyperL- Hyperlipidaemia ,IF- immunofluorescence, AF- atrial fibrillation, TIA- transient ischemic attack, DM- Diabetes mellitus, GI- gastro- intestinal, CKD-Chronic kidney disease ,MI- myocardial infarction, HF- heart failure, COPD- chronic obstructive pulmonary disease, Cr – Creatinine , ARDS- Acute respiratory distress syndrome, HRCT- High-resolution computer tomography, CYC- cyclophosphamide, PEX- plasma exchange or Plasmapheresis , HCQ- Hydroxychloroquine, ABX – Antibiotics.

### Large Vessel Vasculitis

There have been occasional reports of large vessel vasculitis including central nervous system vasculitis occurring with severe COVID-19 where imaging demonstrated a pattern resembling GCA, but these were uncommon[91, 92, 95]. We found no cases of Takayasu’s vasculitis following COVID-19.

### Medium and Small Vessel Vasculitis- ANCA associated vasculitis

The diagnosis of new ANCA associated vasculitis may be challenging in the context of severe COVID-19 infection due to shared anatomical territories of infection and inflammation. Also, the multisystem nature of severe COVID-19 infection and the fact that a multiplicity of autoantibodies, including anti-neutrophil cytoplasmic antibodies (ANCA) may appear during infection may complicate the diagnosis[96]. One potential mechanism for ANCA development and other autoantibody development in general in the context of COVID-19 infection is the presence of extensive neutrophil infiltration and neutrophil extracellular traps (NETs) at sites of immunothrombosis and tissue necrosis that could contribute to tissue tolerance failure with antibodies formation[96–98]. In one report about the prevalence of ANCA in hospitalized COVID-19 patients, 57% of randomly selected patients sera confirmed positive for ANCAs, of which 72% were C-ANCA, and 28% were P-ANCA, but only one sample was myeloperoxidase positive[99]. In the same report, there was a positive association between C-ANCA status with intensive care unit admission but not death. Whether these ANCA autoantibodies play a true role in the pathogenesis and severity of COVID-19 still need to be determined. Also, a plethora of other antibodies have been reported in severe COVID-19 and may reflect tissue tolerance breakdown or occasionally perhaps play a role in pathology[100, 101].

Allowing for these caveats, to the best of our knowledge, there are only six described cases of ANCA associated vasculitis with temporal association with COVID-19 infection; most patients were previously healthy, two females and four males, with an age range from 25 until 64 years. All described cases were ANCA positive, and the diagnosis made simultaneously or shortly after the COVID-19 diagnosis. All patients survived after treatment with immunosuppressive medications with some accruing irreversible end organ damage [83–87].

### Immunoglobulin A Vasculitis (IgAV)/Henoch-Schönlein Purpura

IgAV is typically a childhood and early adolescent disease and is linked strongly to triggering environmental factors with subsequent immune-complex deposition and complement activation [102]. Sixteen cases of IgAV were described in correlation with COVID-19 infections, some of the cases presented simultaneously with COVID-19, but others appeared more than one month after the infection, the majority were males, and the age range was between 1 to 78 years. A high percentage of the patients suffered from purpuric rash and gastrointestinal symptoms. In some cases, the diagnosis was confirmed by skin or renal biopsy; skin biopsies revealed the classical finding of leukocytoclastic vasculitis, but some lacked positive IgA staining [89]. A Kidney biopsy was reported in six patients, all kidney biopsies were positive for IgA on direct immunofluorescence staining, usually in the context of a mesangial proliferative pathology [88–90]. Electron microscopy showed mesangial and subendothelial immune deposits to podocyte effacement in IgAV [88–90].

### Goodpasture's syndrome

**Fig. 3:**
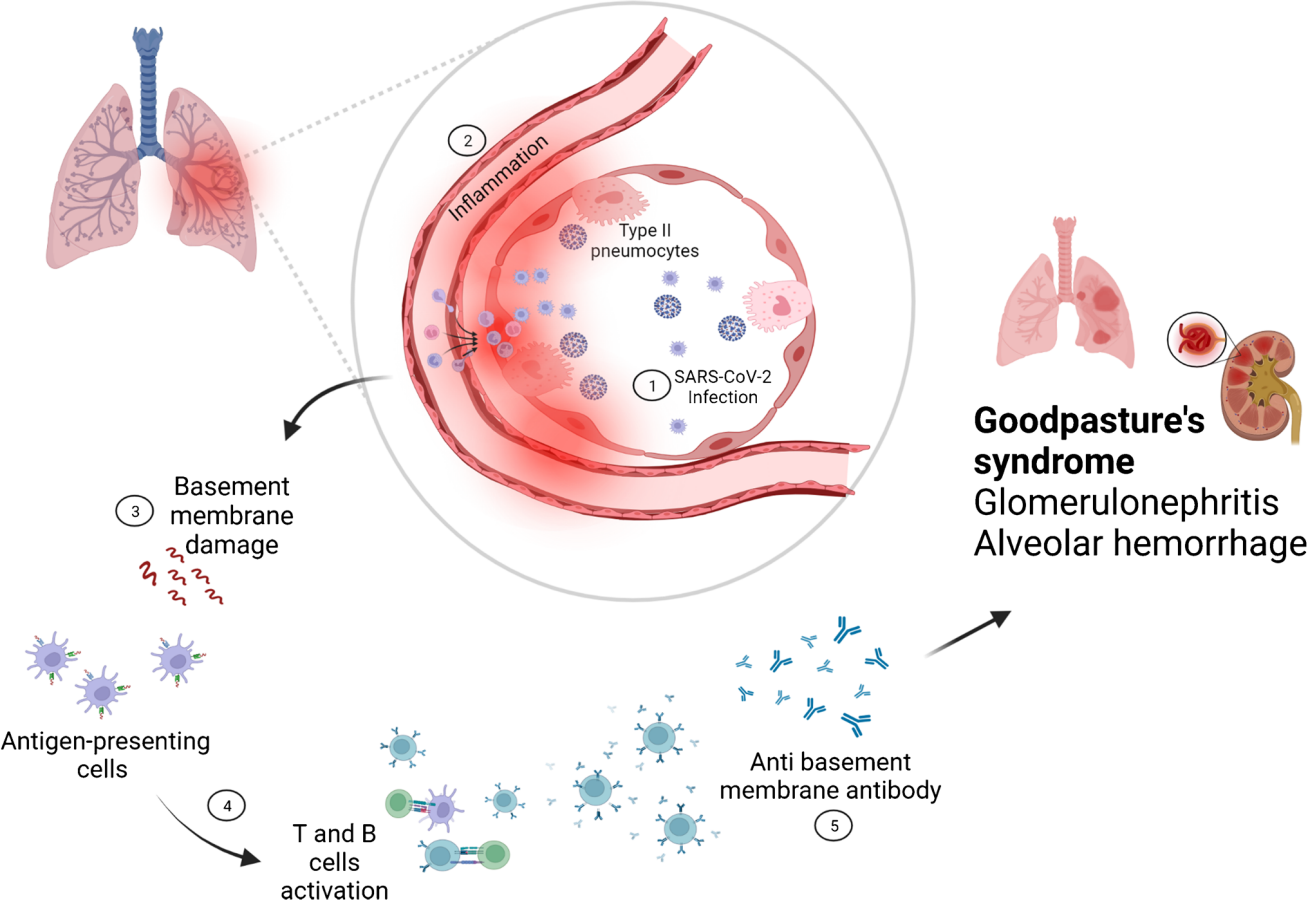
COVID-19 infection triggering Goodpasture's syndrome. SARS-CoV-2 infection triggers profound inflammatory response and basement membrane damage, leading to tolerance failure, which may lead to the production of anti-basement membrane antibodies, eventually resulting in Goodpasture's syndrome

Given that the alveolus and the endothelium are prominent targets in severe COVID-19, then a genuine concern exists that severe SARS-CoV-2 infection with pulmonary basement membrane damage could lead to tolerance failure and the development of autoimmunity (Fig. 3). Indeed, autoimmunity to a plethora of antigens including cytokines and endothelial molecules and others are well reported in severe or critical COVID-19[103, 104]. An interesting case report supplemented by epidemiological data trends in the London region of the UK showed cases of Goodpasture's syndrome increasing following COVID-19 [93]. Thankfully, Goodpasture's syndrome cases in the literature have totalled only nine cases, and it is interesting why this is comparatively underrepresented given the target tissue and the multiplicity of other reported autoantibodies.

## The SARS-CoV-2 Virus Bespoke Vasculitides

Although not included in our systemic literature review, “COVID toes” and KD like vasculitis were the most frequently reported vasculitides with COVID-19 infection and discussed separately below**.** Otherwise, healthy people presented during the first phase of the COVID-19 outbreak without respiratory symptoms and SARS-CoV-2 PCR test negativity but with chilblain lesions, of erythema or dusky discolouration of the digits in particular[105–107]. Histological evaluation of these lesions confirmed a small vessel vasculitis with lymphocytic infiltration and interferon pathway activation within tissue as evidenced by MX-1 staining[108] suggesting a interferonpathy disease mechanism akin to monogenic systemic lupus erythematosus variants as first defined by Crow et al.[109, 110]. These lesions were identical to the typical chilblain pattern of autoimmune chilblain lesions exhibiting both a similar pattern of lymphocytic infiltration and interferon staining[111, 112].

A severe inflammatory reaction termed MIS has been reported in children and encompasses cardiac aneurysmal involvement [113–116], and it seems to be the most common reported vasculitis syndrome after SARS-CoV-2 infection with estimated incidence of 316 person per 1,000,000 SARS-CoV-2 infection in person younger than 21 years and most closely resembles KD[117, 118]. Prior to the emergence of SARS-CoV-2 this vasculitic pathology was a typical feature of KD in children under five of age but the emergent SARS-CoV-2 related pathology affects older children who develop MIS [119, 120]. Unlike COVID-19 pneumonia, young subjects who develop MIS may be a PCR negative for SARS-CoV-2 but usually antibody positive [113, 118, 120–122], suggesting some sort of immune hypersensitivity reaction to SARS-CoV-2. Beyond the KD-like vasculitis, patients may also experience prominent abdominal symptoms[121] and different mechanisms have been advocated to explain this genuine vasculitis related pathology. Given that KD itself is poorly understood, it remains unclear how KD like disease develops with different theories including potentially a superantigen mediated disease related to persistent spike protein antigen [123].

## SARS-CoV-2 Vaccination Reports of Vasculitis.

There is a well-recognized association between natural infection or vaccination and the occasional precipitation or activation of either autoinflammatory or autoimmune diseases, but causality is not certain. Given the known role of spike protein potentially downregulating ACE in experimental settings then a theoretical concern exists around spike protein-based coronavirus vaccines and vasculitis pathology [20, 124]. This is mitigated against by very low level of systemic protein expression with vaccines based on RNA backbones[125]. A few well documented rare immune reactions have been described following SARS-CoV-2 vaccination, most notably vaccine-induced immune thrombotic thrombocytopenia (VITT) after DNA vaccination and myocarditis following RNA vaccination [126–130]. Furthermore, spike protein-based vaccines permit investigation of whether a specific viral component, namely spike protein, might be linked to recognized immunopathology such as KD like disease or other vasculitis.

As of September 2021, there were fifty cases [131–151] of reported vasculitis with temporal association with COVID-19 vaccination. Of these cases, only twenty-three peer-reviewed cases are newly diagnosed vasculitis (Table 2) [131–147]. Cutaneous vasculitides were the most common type of reported vasculitis, with a total of ten patients described in the literature[131–138], and noting that we excluded “COVID toes” from our search these included cutaneous small-vessel vasculitis, leukocytoclastic vasculitis and urticarial vasculitis. This was followed by ANCA associated vasculitis with total of five patients[139–142, 144] and then with IgA vasculitis or HSP with four reported cases[143–146]. Eight cases had underlying comorbidities. Only three of these cases had prior SARS-CoV-2 infection. Symptom onset occurred from a few hours to four weeks after COVID-19 vaccination, all patients had a good outcome, and full recovery was generally reported.

**Table 2:** Reported cases of genuine vasculitis after COVID-19 vaccination

	N.of cases	Gender	Age	Underlying conditions	Symptoms	Workup	Type of vaccine	Treatment
Cutaneous: CSVV [131–134]/LCKV[135–137]/ UV[136, 138]	CSVV:4 LCKV: 4 UA: 2	3 X M 5 X F 2 × N/A	31–83	Only in two patients: 1.HTN; HyperL; mechanical AVR (on warfarin); Algy to ibuprofen (mild rash) 2.HTN; Hypothyroidism	CSVV: Purpuric rash; fever; Itchy maculo-papular rash; pitting oedema LCKV: Purpura UV: Urticarial rash; fever; arthralgia	Clinical; CRP + ESR + ; auto immune serology + ; skin biopsy	1 X Jan 1 X Oxf 3X Pfi 1X COVAXIN®; 2XMod 1X Whole Virion inactivated 1XN/A	CSVV: Systemic or topical steroids /anti Histamine LCKV: NA in two cases. Sys ABX and Topical Steroids in one case UV: Oral indomethacin, topical calamine lotion, levocetirizine Antihistamines, steroids, dapsone
ANCA-associated vasculitis[139–142, 144]	5	2XF 3XM	37–81	Only in two patients: 1. Graves’ disease 2. T2DM; HTN;PAF	Flu-like; Anorexia; Rash; fever; pain; Haemoptysis; GI symptoms	Clinical; ANCA + ; CRP + ; Cr + ; Proteinuria; Haematuria; Skin Biopsy Renal biopsy (in two cases); Chest CT scan; [18F]FDG-PET/CT (increased uptake in middle-sized vessels)	2X Pfi 2X Oxf 1XMod	Sys steroids, oral CYC/ Rituximab/plasmapheresis if needed
IgAV [143, 144]/HSP[145, 146]	IgAV:2 HSP:2	IgAV:2XM HSP:2XF	39–72	HTN; MI;T2DM; obesity; asthma. Osteosarcoma; intercostal shingles; tonsillectomy Hashimoto’s thyroiditis; Assisted reproductive therapy	Flu-like illness; Purpura; Arthralgia; Fever; macroscopic haematuria	IgAV: Clinical; CRP + ; Cr + ; Skin biopsy one case; Renal biopsy in one case; HSP: Clinical; CRP + ; ANA + , RF + ; Microscopic haematuria	2XOxf 1XMod 1XPfi	IgAV: Sys steroids, CYC when needed HSP: Sys steroids
Vasculitis NOS[135]	3	N/A	N/A	N/A	N/A	N/A	2xMod 1XPfi	N/A
LVV [147]	1	F	78	None	Cephalalgia, Osteomyalgia	[18F] FDG-PET/CT + (large arteries of the legs); CRP + , ESR +	Mod	N/A

Abbreviation: CSVV- Cutaneous small-vessel vasculitis, LKCV- Leukocytoclastic vasculitis, UV- Urticarial vasculitis ,IgAV- IgA Vasculitis, HSP- Henoch-Schönlein Purpura, ANCA -Antineutrophil cytoplasmic antibodies, LVV – Large vessel vasculitis , + - positive/elevated, F- Female, M- Male, HTN- Hypertension ,HyperL- Hyperlipidemia ,AVR -Aortic valve replacement ,Algy – Allergy ,Mod -Moderna COVID-19 vaccine (mRNA-1273),Jan- Janssen Ad26.COV2.S, Oxf- Oxford-AstraZeneca, Pfi- BNT162B2/Pfizer, Sys- systemic ,PAF- paroxysmal atrial fibrillation, T2DM- Type 2 Diabetes mellitus, GI- gastro- intestinal ,Cr – Creatinine, RF- rheumatoid factor, CT- computer tomography, CYC- cyclophosphamide , ABX – Antibiotics, N/A-not available, NOS-not otherwise specified

Despite anecdotal reports of COVID-19 vaccine-induced MIS, larger cohorts have not confirmed as association. In one report of 20 patients with MIS, seven reported to have MIS after COVID-19 vaccination; all patients had evidence of previous COVID-19 infection, so COVID-19 induced MIS was not ruled out in these cases[152]. In another report of 107 pediatric patients admitted to intensive care units in France within two months period because of MIS, 33 of them were eligible for vaccination but none were fully vaccinated, and only 7 received one dose [135, 153]. It remains uncertain whether spike protein vaccines alone can trigger MIS, thus incriminating this antigen that MIS.

The cutaneous “COVID toes” has been reported in temporal association with COVID-19 vaccination, which likely reflects poorly understood excessive type 1 interferon response to spike protein encoding nucleic acid and is reminiscent of SARS-CoV-2 where replicating virus is not usually detectable in COVID toes [154, 155].

As stated earlier, Goodpasture syndrome was associated with the COVID-19 pandemic[93] With respect to relevant vaccination, only two cases of questionable anti-GBM nephritis without pulmonary involvement were reported. In the first case, anti-GBM antibodies were negative, and the diagnosis was suspected based on histology[156]. In the putative second case anti-GBM nephritis was accompanied by mesangial IgA deposit [157]. In some way, this difference in Goodpasture frequency may be explained by the absence of tissue damage after the COVID-19 vaccination as opposed to SARS-CoV-2 infection in which basement membrane damage provokes the production of anti-basement membrane antibodies but case numbers are too small to be definitive (Fig. 3).

## Potential COVID Vasculitis Mimic Related to Small Vessel Thrombosis

As distinct from the vasculitis occurring in mild COVID-19 disease that is IFN associated, a second clinical pattern of cutaneous vasculitis has been reported in severe or critical COVID-19. It is possible that some of the aforementioned diagnosed vasculitides may in fact fall within the vasculitis mimic category, that is linked to primary immunothrombotic mechanisms. Retiform purpura, livedo reticularis and purpuric lesions have a quite distinct immunopathology and are characterized by vessel lumen thrombosis and pauci-immune infiltration and an absence of IFN pathway protein expression in the tissue[105, 108, 158, 159].

Akin to lung pathology in COVID-19 pneumonia, there is a lack of evidence for direct endothelial infection. However, immunochemistry has shown staining for SARS-CoV-2 related proteins in the vascular lumen[32, 159–161]. It has been suggested that such pulmonary shedding of SARS-CoV-2 non-replicating viral fragments or “pseudo-virus” (spike, envelope, membrane proteins with or without viral RNA) may lead to ACE-2 engagement with resultant endothelial disturbance triggering local inflammation and then the wider thrombotic and vasculitic pathology[162]. It has also emerged that viral protein antigenaemia is strongly linked to systemic inflammatory responses and severity of COVID-19 pneumonia. These pauci immune lesions have been associated with complement C5-9 terminal pathway activation. Whether complement is playing a role driver role in such thrombotic immunopathology is unclear as vascular ischemia damage may also activate the complement system[40, 159, 163–166].

**Fig. 4:**
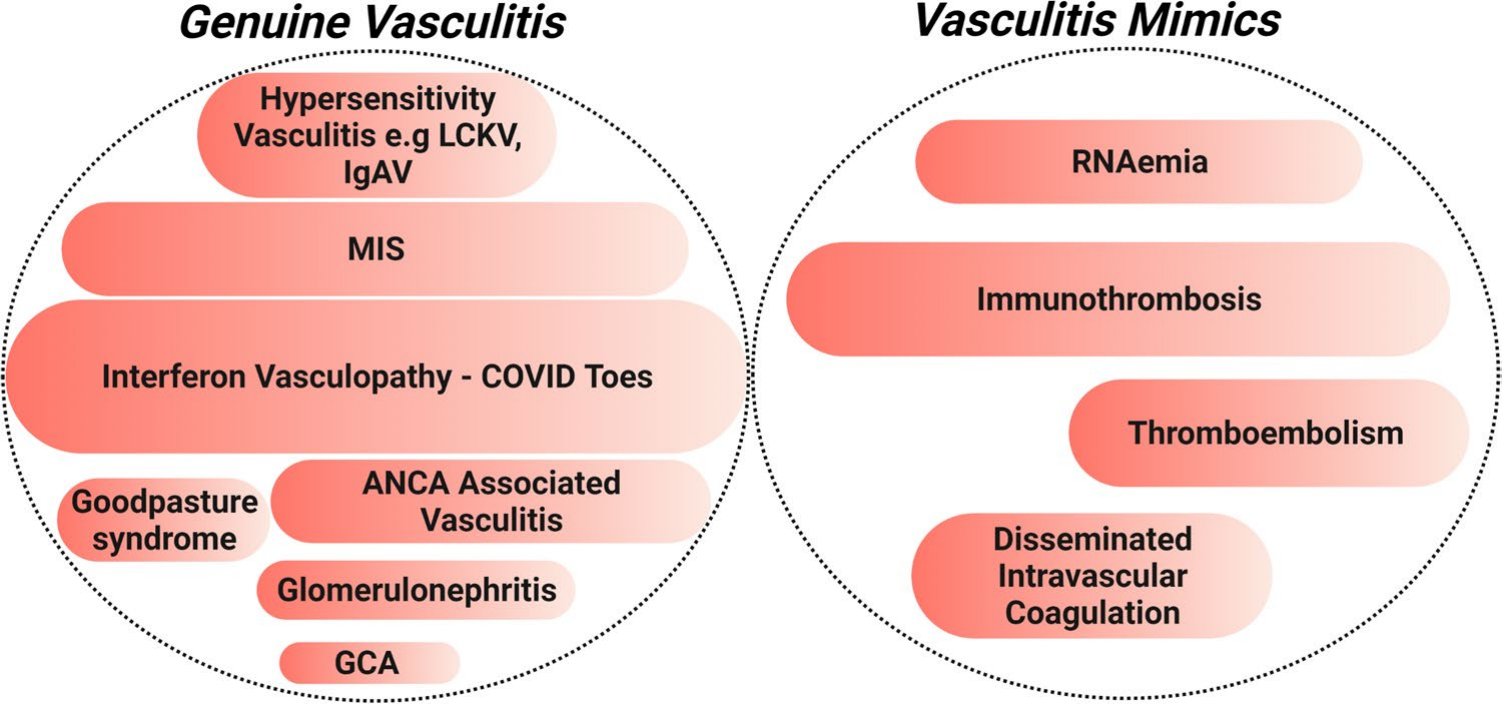
The COVID-19 vasculopathy spectrum. Abbreviations: LCKV-Leukocytoclastic vasculitis, IgAV- IgA Vasculitis, ANCA- Antineutrophil Cytoplasmic Antibodies, GCA- Giant cell arteritis, MIS- Multisystem inflammatory syndrome

We have proposed two simple mechanism for this vasculitis mimic immunopathology[167]. First, physiological immunothrombosis contains the SARS-CoV-2 virus in the pulmonary territory but breakdown of the alveolar-vascular barrier with translocation of viral RNA and debris to the systemic circulation including small arteriolar, capillary, and venous circulation is associated with RNAaemia activation of immunothrombosis at sites distant from the lungs (Fig. 4). Given that the extensive pulmonary immunothrombosis is also evident in the pulmonary venular territory in postmortem studies and occasionally thrombosis in the pulmonary vein root on computed tomography pulmonary angiogram studies, we proposed that embolization from this territory distal to the alveolar-capillary clot filtration system offered a novel hitherto unappreciated source of embolization[168, 169]. Further detailed postmortem pathological studies of lung tissue and distal organs are needed to confirm this theory.

Critical COVID-19 pneumonia is associated with organ ischaemia in many territories including the brain where cryptogenic strokes are commonly reported[170, 171]. Some of these ischemic complications, especially pulmonary embolism and venous thrombosis are much commoner with COVID-19 compared to influenza A pneumonia and likely reflects the differential alveolar versus bronchial tissue tropism[172–174]. These lesions do not show typical histological features of vasculitis and are pauci immune. Haematuria is reported in over 40% of cases with renal involvement in severe COVID-19 pneumonia but histologically the changes are of ischaemic injury rather than vasculitis[175, 176]. Likewise, intestinal ischemia is a common manifestation and histology shows diffuse ischemic change rather than vasculitis[177]

## Conclusions

The massive alveolar-capillary territory juxtaposition forms the key insight to understanding the physiological role of vascular immunity in containing infection within the alveolar space. This is associated with extensive non-infectious endotheliitis or endothelialopathy that appears to be predominantly cytokine and inflammatory mediator driven and dysregulation of this pathway leads to local and systemic immunothrombosis that serves as a vasculitis mimic. Despite the initial obvious link with the ACE2 receptor, it appears that the vasculitis mimic mechanism is not strongly linked to this. Occasional vasculitis does occur with COVID-19 and with the exception of the rare MIS pathology the vasculitis is usually self-limiting and furthermore both KD like vasculitis and “COVID toes” do not appear to be linked to productive viral infection in airways or the lungs. Mechanistically, COVID toes have been best conceptualized in relationship to excessive interferon driven response in the skin with strong similarities to the monogenic type 1 interferonopathies that caused chilblain lupus[108]. MIS exhibits features of KD in some patients including coronary vasculitis, but the mechanism remains unclear as KD itself is not well understood. There is no evidence that SARS-CoV-2 vaccine strategies, many of which encode for spike protein, have contributed to the bone fide vasculitis pathology apart from occasional reports of “COVID toes” following vaccination. Overall, the COVID-19 pandemic has served a highly informative model for a refined understanding of vascular immunopathology mechanism following the global emergence of a novel RNA virus.
